# Community Collectivization and Consistent Condom Use Among Female Sex Workers in Southern India: Evidence from Two Rounds of Behavioral Tracking Surveys

**DOI:** 10.1007/s10461-015-1171-2

**Published:** 2015-08-19

**Authors:** Shanti Vejella, Sangram Kishor Patel, Niranjan Saggurti, Parimi Prabhakar

**Affiliations:** India HIV/AIDS Alliance, Sarovar Center, Secretariat Road, Hyderabad, India; HIV and AIDS Program, Population Council, 142, Golf Links, New Delhi, 110003 India

**Keywords:** Community collectivization, Consistent condom use, Female sex worker, Mediation analysis, Interaction effect, Andhra Pradesh, India

## Abstract

Community collectivization is an integral part of condom use and HIV risk reduction interventions among key population. This study assesses community collectivization among female sex workers (FSWs), and explores its relationship with sex workers’ consistent condom use (CCU) with different partners considering the interaction effect of time and collectivization. Data were drawn from two rounds of cross-sectional surveys collected during 2010 (N1 = 1986) and 2012 (N2 = 1973) among FSWs in Andhra Pradesh, India. Results of the multiple logistic regression analysis show that, CCU with regular and occasional clients increased over the inter-survey period among FSWs with a high collective efficacy (AOR 2.9 and 6.1) and collective agency (AOR 14.4 and 19.0) respectively. The association of high levels of collectivization with CCU and self-efficacy for condom use are central to improve the usefulness and sustainability of HIV prevention programs worldwide.

## Resumen

Una de las partes esenciales del uso del condón y de reducción de riesgos de VIH población clave es la Colectivización Comunitaria. Esta investigación evaluará la colectivización comunitaria entre las trabajadoras sexuales y explorará su relación con el uso constante del condón con parejas diferentes por la parte de las trabajadoras sexuales, teniendo en cuenta el efecto de la interacción, de la duración. Se ha extraido los datos de una encuesta transversal de dos rondas, recogidos durante 2010 (N1 = 1986) y 2012 (N2 = 1973) entre las trabajadoras sexuales en Andhra Pradesh, India. Los resultados de la regresión logística múltiple muestran que el uso constante de condón con clientes regulares como ocasionales aumentó durante el período de inter-encuesta entre las trabajadoras sexuales con un alto eficacia colectiva (AOR: 2.9 y 6.1) y agencia colectiva (AOR: 14,4 y 19.0) respectivamente. La conexción entre altos niveles de colectivización con el uso constante del condón y auto-eficacia para el uso del condón, son esenciales para mejorar la utilidad y la sostenibilidad de los programas de prevención del VIH, en todo el mundo.

## Introduction

Addressing the HIV risk and vulnerability of key populations needs unique approaches. Programs addressing the HIV risk of female sex workers (FSWs) and other key populations need to focus on both individual risk behaviors and social-structural factors (stigma, discrimination, disempowerment, violence and socio-economic marginalization) that shape the context of risk [1]. Over the years, international agencies and governments have implemented programs to reduce stigma and discrimination among persons living with HIV (PLHIV) and key populations, and promoted prevention strategies to combat the HIV/AIDS epidemic [2]. Globally, studies have documented that community-led HIV prevention interventions for FSWs are associated with increased knowledge of HIV risk [3], increased condom use with clients and partners, [4–6] and decreased prevalence of sexually transmitted infections (STIs) [3, 6]. These community-led structural interventions are important in changing the risky behavior of social and physical environment of FSWs [7–9]. Structural interventions under Avahan, the India AIDS Initiative of the Bill & Melinda Gates Foundation (BMGF), are defined as interventions addressing social, economic, and political issues that affect health at the individual, community, and societal levels [10]. In addition, structural interventions encourage HIV prevention activities by addressing these environments, increasing the availability of behavioral choices and addressing the barriers to behavior change [11].

Community mobilization is an integral part of structural interventions for HIV risk reduction [11–16]. Over the years, community mobilization has been defined and operationalized in different ways [7, 17–20]. The Avahan program in India describes community mobilization as a process by which key populations ‘‘utilize their intimate knowledge of vulnerability to overcome the barriers they face and realize reduced HIV risk and greater self-reliance through their collective action’’ [10]. Further, community mobilization (collectivization) aims not only to empower key populations as a group to reduce vulnerability, but also to improve their self-efficacy (defined as the ability to control and make decisions about one’s own behaviors), which ultimately influences the adoption and maintenance of healthy behaviors [20–23]. The Sonagachi project in Kolkata [24] and the Mysore project in Karnataka [25] are successful models of community-led structural interventions among FSWs in India. In fact, the lessons on community mobilization learned from the Sonagachi project have largely influenced the implementation of the Avahan program in six high HIV prevalence states of India [21], and one of the objectives of the Avahan program is to mobilize key populations to manage and implement HIV prevention programs [10, 26].

The importance of community mobilization based structural interventions in HIV prevention programs for sex workers has been documented worldwide as well as in India [4, 20, 27–30]. Standard community mobilization indicators of collective efficacy, collective agency and collective action have generally been used in HIV prevention studies in India and elsewhere [19, 20, 29, 31]. However, further investigation is needed on the association between community mobilization and safer sexual behavior among sex workers [5, 16, 32]. According to the Centers for Disease Control and Prevention (CDC), nearly 70 % of HIV infections can be reduced by CCU among key populations [33]. Taking these points forward, it is important to know the relationship between community collectivization, self-efficacy and consistent condom use (CCU) among FSWs in the Indian context. This study assesses the degree of collectivization among FSWs in Andhra Pradesh, a high HIV prevalence state in southern India over the two time periods 2010 and 2012, and explores its relationship with FSWs’ CCU behavior, self-efficacy for condom use with commercial clients and their interaction effects. We also examine the mediating effects of FSWs’ self-efficacy on condom use with clients by the degree of collectivization during 2010 and 2012.

## Materials and Methods

### Data

This paper uses two rounds of data from the behavioral tracking survey (BTS), a cross-sectional survey conducted in 2010 (BTS-I) and 2012 (BTS-II) among FSWs in Andhra Pradesh. The BTS was conducted once in 2 years on a sample population to track the behavioral outcomes over time under the Avahan program. The information obtained through these regular field-based surveys is used to both track the progress of the program, and make mid-course corrections, as needed. The survey monitors critical components of the program, including community mobilization, condom promotion, STI management, behavior change communication, sustainability and advocacy. FSWs were recruited into the survey from five program districts (Khammam, Warangal, Kurnool, Medak, Ananthapur), selected from a total of 7 Avahan program implementation districts in Andhra Pradesh. A sample size of 400 FSWs was calculated for each district based on the prevalence of CCU and expected level of change with each unit change in the degree of community mobilization.

In both the survey rounds, a uniform sampling design was followed, in which the sampling frame was prepared to select FSWs from each hot spot (place where FSWs congregate to solicit clients), after a rapid mapping exercise that was conducted using key informant interviews with local community members, police staff and social workers. The sampling frame prepared through such an exercise validated the existing list of hot spots originally developed by the program-implementing agency. The hot spots were then grouped into two categories: (1) non-public (brothels, hotels, lodges, roadside cafes, and homes), and (2) public (streets, market areas, highways, and cinemas). A probability sampling method was used to select respondents. Conventional cluster sampling was used for non-public hot spots and time location cluster (TLC) sampling for public hot spots [34]. The TLC method involved dividing a hot spot into several clusters based on the time slots (e.g., 5 pm–9 pm) when FSWs gathered at the hot spot, and then randomly selecting the required number of clusters. In the second stage, respondents were randomly selected within each selected hot spot. A total sample of 1986 FSWs was collected during BTS-I (2010), while a sample of 1973 FSWs was collected in BTS-II (2012) (see Table 1). All interviews were conducted by trained female interviewers with verbal and written skills in Telugu, the local language of Andhra Pradesh. The survey questionnaire was developed in English and translated into Telugu. The translated forms were reviewed by study investigators fluent in both languages. The interview schedule was pre-tested in communities similar to the survey sites. All the interviews were held in a private location specifically hired for the survey or in a location convenient to the study participants. Field staff checked the data immediately after the interviews to ensure accuracy and completion of the questionnaire. A user-written computer program in CSPro (version.4.0) was used for double data entry by trained data entry officers.

**Table 1:** Socio-demographic characteristics of female sex workers (FSWs), Andhra Pradesh, India, behavioral tracking survey-I (2010) and II (2012)

Background characteristics	Percentages and mean (SD)		
	BTS I (2010)	BTS II (2012)	*p* Value
Age	29.2 (5.3)	29.3 (5.7)	
Age			0.003
<30 years	53.3	52.0	
≥30 years	46.7	48.0	
Marital status			0.000
Never married	9.2	6.6	
Currently married	57.1	50.0	
Widowed/deserted/separated/divorced	33.7	43.4	
Education			0.000
No formal education	44.4	62.3	
Having formal education	55.6	37.7	
Main source of solicitation			0.000
Brothel/lodge	5.9	6.6	
Street/public places	46.8	40.6	
Home	10.0	5.1	
Mobile phones	31.8	42.7	
Others	5.6	4.9	
Currently under debt			0.271
No	14.7	20.0	
Yes	85.3	80.0	
Source of income other than sex work			0.000
Sex work only	22.0	30.8	
Sell vegetable/flower	14.6	8.6	
Work as daily laborer	41.6	47.0	
Work as domestic help	11.0	4.0	
Salaried employee	2.3	2.1	
Others	8.6	7.5	
Mobility for sex work			0.000
No	62.0	73.2	
Visited places and had sex in last 2 years	38.0	26.8	
Average duration of practicing sex work (in years)	4.4 (2.4)	4.8 (3.3)	
	N1 = 1986	N2 = 1973	

*BTS* behavioral tracking survey; p Value were calculated through *χ* ^2^test

### Ethical Considerations

The study design and questionnaires were approved by the institutional review boards of Family Health International and the Karnataka Health Promotion Trust. The survey instruments were developed, piloted and revised through a community consultation process, which was supported by the participating institutions. During the survey, peer educators (sex worker community) from the local areas and outreach workers (community member) from the program at block level were identified in each district, who were briefed the FSWs about the survey. Verbal consent was obtained from all respondents prior to participation in the survey. For ethical reasons, in both the rounds of BTS, females 18 years or above who had sex in exchange for cash/kind in the last 1 month were identified as FSWs and the information was collected accordingly. No names and addresses were recorded on the questionnaires. Participants were not provided any compensation for their time in the study but were referred to local project sites run by the implementing agency in the survey districts for more information and services.

In this survey, a community advisory board was not constituted; however, before and after completion of the survey, community-based and/or civil society organizations in the respective districts were informed about the survey process, objectives and challenges. The survey findings were disseminated to the multiple stakeholders, including community members, program implementing partners and Andhra Pradesh State AIDS Control Society (APSACS) officials. Sex workers participated and made some of the presentations on key findings in the local language during dissemination meetings and the findings were well received by the community members.

### Measures

The socio-demographic variables considered in the analysis were age; formal education (yes, no); marital status (never married, currently married, and formerly married); no source of income other than sex work (yes, no); duration of sex work; mobility for sex work within and/or outside district in past 2 years (yes, no); currently under debt (yes, no); and place of solicitation for sex (brothels/lodges, homes, mobile phones, and street/public places). Three independent variables comprising community collectivization indictors were considered in the analysis: collective efficacy, collective agency and collective action; each of these variables was made up of multiple indicators comprising a composite index described in detail in the following section.

#### Community Collectivization Indicators

Collective efficacy refers to FSWs’ belief in the power of the community to work together to bring about positive change. This was measured based on responses to the question: How confident are you that FSWs in your community can work together to achieve the following goals: keep each other safe from harm; increase condom use with clients; speak up for your rights; and improve your lives? Responses to these questions included: 1 = not at all, 2 = somewhat, 3 = very and 4 = completely confident. Using these four questions and corresponding responses, an index was constructed, with the scale values ranging from 1 to 4, which had a reliability (Cronbach’s alpha) of 0.821. The index score was further divided into two equal categories of collective efficacy: 0 = low (1–2.4999) and 1 = high (2.5–4).

Collective agency refers to the choice, control and power that FSWs have to act for themselves to claim their rights (whether civil, political, economic, social or cultural) and to hold others accountable for these rights. This indicator was measured based on responses to the question: In the past 6 months, have you negotiated with or stood up against the following stakeholders– police, madam/broker, local goon (gang member), clients or any other sexual partner– in order to help a fellow sex worker or to help fellow sex workers? A separate question for each of the above stakeholders was asked, with the possible binary response categories ‘Yes’ (coded as 1) and ‘No’ (coded as 0). Using these four questions and corresponding responses, an index was constructed, with the scale values ranging from 0 to 1, which had a reliability (Cronbach’s alpha) of 0.889. The index score was further divided into two equal categories of collective agency: 0 = low (0–0.4999) and 1 = high (0.5–1).

Collective action refers to the strategic and organized activities of mobilized community members to increase the community’s visibility and present or enact its agenda for change (for example, through rallies, demonstrations, or meetings with stakeholders). This was measured based on responses to the following seven questions: Whether the sex workers group comes together to demand/help for the following: (1) ration card, (2) voters card, (3) bank account, (4) free education for children, (5) health insurance, (6) representation in government forums, and (7) better health services from the government. A separate question was asked for each of the above social entitlements and services, with the possible binary response categories ‘Yes’ (coded as 1) and ‘No’ (coded as 0). Using these seven questions and corresponding responses, an index was constructed, with the scale values ranging from 0 to 1, which had a reliability (Cronbach’s alpha) of 0.990. The index score was further divided into two equal categories of collective action: 0 = low (0–0.4999) and 1 = high (0.5–1).

#### Outcome and Mediation Indicators

The key outcome indicator used for analysis was CCU with commercial sex partners. CCU with a given type of client (occasional, regular) was defined as the use of condom in every sexual encounter with that type of client. Occasional clients were defined as men whom FSWs did not know or did not recognize their faces. Regular clients were defined as men whom FSWs knew well and could recognize their faces. While examining the association between the degree of collectivization and outcome indicators, we also assessed the role of individual-level efficacy variables as potential mediating factors for indirect benefits of community mobilization. According to Albert Bandura, self-efficacy is “the belief in one’s capabilities to organize and execute the courses of action required to manage prospective situations [22].” In other words, self-efficacy is a person’s belief in his or her ability to succeed in a particular situation. A similar principle was followed in the Avahan program, which also emphasizes the marginalized population’s self-efficacy based on the social cognitive theory and Bandura’s definition of self-efficacy. In this study, self-efficacy for condom use with commercial sex partners was included as a potential mediator for the relationship between collectivization and CCU with commercial sex partners. Self-efficacy for condom use with commercial sex partners refers to FSWs’ ability to negotiate condom use with their commercial sex partners in certain circumstances. The questions used in the questionnaire are: How confident are you that you can use a condom with each commercial partner when (1) he gets angry with you; (2) he offers you more money for sex without a condom; or (3) you have been using alcohol or drugs? Responses to these questions included: not at all (coded as 1), somewhat (coded as 2), very (coded as 3), and completely confident (coded as 4). Using these four questions and corresponding responses, an index was constructed, with the scale value ranging from 1 to 4, which had a reliability (Cronbach’s alpha) of 0.822. The index score was further divided into two equal categories of self-efficacy for condom use with clients: 0 = low (1–2.49) and 1 = high (2.5–4).

### Data Analysis

The data were analyzed across the two survey rounds using descriptive statistics (i.e., means, standard deviations, and proportions) and bivariate analysis to describe the strength and association between collectivization and the outcome indicators. Adjusted odds ratios (AOR) and their 95 % confidence intervals (CI) were estimated, after adjusting for socio-demographic characteristics, to assess the independent relationship between degree of collectivization and the potential mediators and outcome indicators. The time and collectivization interaction effects were also used to assess the total change in outcome indicators over time. The interaction effect used here can be defined as “the differing effect of one independent variable (collectivization) on the dependent variable (outcome indicators), depending on the particular level of another independent variable (time)” [35]. This concept is useful and common in social and health science research. A significant association between collectivization and the outcome measures was considered to be essential for the mediation analysis [36]. The effect of a collectivization indicator on any outcome indicator was considered to be mediated through a potential mediator if the following conditions were met: (1) collectivization was significantly associated with the outcome indicator, (2) collectivization was significantly associated with the potential mediator, or (3) the relationship between collectivization and the outcome indicator declined when the mediating variable was entered into the regression model [36, 37]. The first two conditions were assessed by estimating the independent relationship between the collectivization indicators and the outcome indicator and the potential mediating variable. The third condition was evaluated by entering the potential mediating variable as one of the independent variables in the multivariable logistic regression model used to examine the relationships between collectivization and outcome indicator. All analyses described above were conducted using STATA software (version 11.2).

## Results

Table 1 presents a profile of FSWs across the two survey rounds. Almost half (47 and 48 %; 2010 and 2012, respectively) were 30 years or older (with average age of around 29 years); half or more were currently married (57 and 50 %); and those with formal education ranged from 56 to 38 % respectively. Little more than two-fifths solicited clients on the street or in public places (47 vs. 41 %; respectively), followed by mobile phones for solicitation (32 and 43 %; respectively), home-based solicitation (10 and 5 %; respectively), and brothel/lodge-based solicitation (6 and 7 %; respectively). More than one-fifth reported that sex work was their only source of income (22 and 31 %; respectively). Most FSWs were in debt (85 and 80 %; respectively) at the time of survey. The average duration in sex work increased from 4.4 to 4.8 years (2010–2012), while mobility for sex work declined from 38 to 27 % over the same period.

Among the community collectivization indicators, collective efficacy (89 vs. 85 %) and collective agency (51 vs. 42 %) showed a marginal decrease over the inter-survey period (from 2010 to 2012), while collective action (13 vs. 29 %) showed a significant increase (Table 2). The potential mediator, self-efficacy for condom use with clients, increased significantly by 10 % from 2010 (63 %) to 2012 (73 %). Further, the outcome indicator, CCU with occasional clients, increased significantly from 2010 (72 %) to 2012 (85 %). Similarly, CCU with regular clients increased by 13 percentage points (64 % to 76 %) from 2010 to 2012. High degree of collectivization was significantly associated with most of the outcome indicators and potential mediators of condom use over the two survey rounds, even after adjusting for individual background characteristics (Table 3). FSWs who reported a high degree of collective efficacy were more likely than those who reported low levels of collective efficacy to report CCU with occasional clients (2010: 72 vs. 73 %, AOR 1.1, 95 % CI 0.8–1.5; 2012: 59 vs. 90 %, AOR 6.3, 95 % CI 4.5–8.9; Interaction effect: AOR 6.1, 95 % CI 3.8–9.8; p < 0.001); and CCU with regular clients (2010: 60 vs. 65 %, AOR 1.3, 95 % CI 0.9–1.8; 2012: 53 vs. 80 %, AOR 3.5, 95 % CI 2.6–4.8; Interaction effect: AOR 2.9, 95 % CI 1.9–4.5, p < 0.001). FSWs who reported a high degree of collective efficacy were less likely to report a high degree of self-efficacy for condom use with clients than those who reported low levels of collective efficacy (2010: 55 vs. 65 %, AOR 1.5, 95 % CI 1.1–2.1; 2012: 69 vs. 73 %, AOR 1.2, 95 % CI 0.9–1.7; Interaction effect: AOR 0.8, 95 % CI 0.5–1.3, p = 0.438). Over the survey periods, FSWs who reported a high level of collective agency showed a significant increase in CCU with occasional clients (2010: 78 vs. 67 %, AOR 0.6, 95 % CI 0.5–0.8; 2012: 77 vs. 97 %, AOR 8.5, 95 % CI 5.1–14.0; Interaction effect: AOR 14.4, 95 % CI 8.2–25.3, p = 0.000); regular clients (2010: 72 vs. 57 %, AOR 0.6, 95 % CI 0.5–0.8; 2012: 62 vs. 95 %, AOR 10.7, 95 % CI 7.4–15.4; Interaction effect: AOR 19.0, 95 % CI 12.2–29.6, p < 0.001) and high self-efficacy for condom use with clients (2010: 60 vs. 67 %, AOR 1.2, 95 % CI 0.9–1.6; 2012: 62 vs. 87 %, AOR 4.1, 95 % CI 3.1–5.3; Interaction effect: AOR 3.4, 95 % CI 2.3–5.0, p < 0.001). FSWs who reported a high degree of collective action showed a marginal increase in CCU with regular clients as compared to those who reported low levels of collective action, while FSWs who reported a high degree of collective action were less likely to report high self-efficacy for condom use with clients as compared to those with low levels of collective action.

**Table 2:** Distribution of community mobilization indicators among Female Sex Workers (FSWs), Andhra Pradesh, India, Behavioral Tracking Survey-I (2010) and II (2012)

	Behavioral Tracking Survey		
Community mobilization indicators	2010 (N = 1986)	2012 (N = 1973)	p Value
Community collectivization			
Collective efficacy:(H)	89.0 (1768)	85.0 (1671)	0.000
Work together to keep each other safe from harm	65.6 (1303)	68.4 (1349)	0.063
Work together to increase condom usage	87.3 (1733)	81.3 (1603)	0.000
Work together to speaking for sex workers rights	72.0 (1429)	71.4 (1406)	0.637
Coming together for improving lives of sex workers	61.6 (1223)	72.9 (1437)	0.000
Collective agency:(H)	50.7 (1006)	42.3 (835)	0.000
Negotiated or stood up against police	45.0 (895)	43.8 (864)	0.447
Negotiated or stood up against madam/broker	51.0 (1013)	40.1 (791)	0.000
Negotiated or stood up against local goon	17.5 (348)	39.1 (771)	0.000
Negotiated or stood up against client/regular partner/other partner	42.7 (849)	40.8 (803)	0.225
Collective action:(H)	12.7 (253)	28.5 (563)	0.000
Come together to demand/help for ration card	13.0 (258)	30.2 (595)	0.000
Come together to demand/help for voter card	12.4 (247)	29.3 (577)	0.000
Come together to demand/help for bank account	13.3 (264)	28.4 (560)	0.000
Come together to demand/help for free education for children	17.7 (351)	28.0 (553)	0.000
Come together to demand/help for health insurance	20.7 (412)	28.0 (551)	0.000
Come together to demand/help for representation govt. forum	5.2 (103)	19.9 (392)	0.000
Come together to demand/help for better health services from the govt.	15.2 (302)	30.2 (594)	0.000
Potential mediators			
Self-efficacy for condom use with clients	63.4 (1260)	72.5 (1430)	0.000
Self-efficacy for condom use with regular partners	36.2 (716)	43.3 (852)	0.000
Outcome indicators			
CCU with occasional clients	72.3 (1436)	85.3 (1682)	0.000
CCU with regular clients	64.3 (1260)	76.0 (1478)	0.000
CCU with regular partners	15.3 (273)	18.5 (284)	0.007
CCU non regular nonpaying partners	39.0 (187)	57.0 (182)	0.000

*CCU* consistent condom use; p Values were obtained by testing the significance of differences in percentages (Z-test) between groups

**Table 3:** Relationship of collectivization with outcome indicators (consistent condom use with clients (occasional and regular)) and mediators (self-efficacy for condom use with clients) among female sex workers in Andhra Pradesh, BTS-I (2010) and BTS-II (2012)

Collectivization	BTS I (2010)		BTS II (2012)		Time × collectivization	
	%	AOR (95 % CI)	%	AOR (95 % CI)	AOR (95 % CI)	p Value
*Outcome indicators*						
Consistent condom use with occasional clients						
Collective efficacy						
Low	72.0	Ref	59.0	Ref		
High	73.0	1.1 (0.8–1.5)	90.1	6.3 (4.5–8.9)	6.1 (3.8–9.8)	0.000
Collective agency						
Low	78.0	Ref	77.1	Ref		
High	66.7	0.6 (0.5–0.8)	97.0	8.5 (5.1–14.0)	14.4 (8.2–25.3)	0.000
Collective action						
Low	71.4	Ref	84.0	Ref		
High	78.1	1.4 (0.9–2.2)	89.3	1.6 (1.1–2.3)	1.1 (0.6–2.0)	0.635
Consistent condom use with regular clients						
Collective efficacy						
Low	60.0	Ref	52.7	Ref		
High	65.0	1.3 (0.9–1.8)	80.1	3.5 (2.6–4.8)	2.9 (1.9–4.5)	0.000
Collective agency						
Low	71.7	Ref	62.1	Ref		
High	57.3	0.6 (0.5–0.8)	94.5	10.7 (7.4–15.4)	19.0 (12.2–29.6)	0.000
Collective action						
Low	63.7	Ref	72.8	Ref		
High	68.3	1.2 (0.8–1.9)	84.0	1.9 (1.4–2.5)	1.6 (0.9–2.7)	0.083
*Potential mediators*						
High self-efficacy for condom use with clients						
Collective efficacy						
Low	55.0	Ref	69.2	Ref		
High	64.5	1.5 (1.1–2.1)	73.1	1.2 (0.9–1.7)	0.8 (0.5–1.3)	0.438
Collective agency						
Low	60.3	Ref	62.1	Ref		
High	66.5	1.2 (0.9–1.6)	86.7	4.1 (3.1–5.3)	3.4 (2.3–5.0)	0.000
Collective action						
Low	61.8	Ref	73.6	Ref		
High	74.4	1.8 (1.2–2.9)	70.0	0.7 (0.6–1.0)	0.4 (0.3–0.7)	0.001

*AOR* adjusted odds ratios, *Ref* reference variable, *CI* confidence intervals. Odds ratios were adjusted for age of *FSW* formal schooling (yes, no); marital status (currently married, not currently married); source of income other than sex work (yes, no); place of solicitation for sex work (home, public places, brothel/lodges); visited any place for sex work in past 2 years (yes, no); duration of sex work in years (entered as continuous variable)

Table 4 presents results of the mediation analysis. As seen in the table, in most instances collectivization has a significant impact on the outcome indicators, even after adjusting for the effect of corresponding potential mediating and socio-demographic characteristics. FSWs’ collective efficacy mediated the effect of self-efficacy for condom use with both occasional clients (2010: AOR 0.9, 95 % CI 0.7–1.4; 2012: AOR 6.3, 95 % CI 4.5–11.0; Interaction effect: AOR 6.8, 95 % CI 4.3–11.0, p < 0.001) and regular clients (2010: AOR 1.2, 95 % CI 0.9–1.6; 2012: AOR 3.5, 95 % CI 2.6–4.8; Interaction effect: AOR 3.1, 95 % CI 2.0–4.9, p = 0.001) across the survey period. FSWs’ collective agency significantly mediated the effect of self-efficacy for condom use with both occasional clients (2010: AOR 0.5, 95 % CI 0.4–0.7; 2012: AOR 8.0, 95 % CI 4.7–13.6; Interaction effect: AOR 15.0, 95 % CI 8.2–27.0, p < 0.001) and regular clients (2010: AOR 0.5, 95 % CI 0.4–0.7; 2012: AOR 10.2, 95 % CI 6.9–15.2; Interaction effect: AOR 20.1, 95 % CI 12.6–32.3, p < 0.001) across the survey period. Results from the mediation analysis further suggest that the FSWs’ high degree of mediator (self-efficacy for condom use) had a negative association with the outcome indicators in all instances, even after adjusting for the effect of corresponding collectivization indicators and socio-demographic characteristics. The magnitude of the interaction effect for the mediating factors reduced from round 1 to round 2 of the survey.

**Table 4:** Effects of collectivization and mediators on outcome indicators (consistent condom use with clients) among female sex workers in Andhra Pradesh, BTS-I (2010) and BTS-II (2012)

	BTS I (2010)	BTS II (2012)	Time × Collectivization		BTS I (2010)	BTS II (2012)	Time × Collectivization	
Collectivisation indicators and corresponding mediators	AOR for collectivization^a^ (95 % CI)	AOR for collectivization^a^ (95 % CI)	AOR for collectivization^a^ (95 % CI)	p-value	AOR for mediators^b^ (95 % CI)	AOR for mediators^b^ (95 % CI)	AOR for mediators^b^ (95 % CI)	p-value
*Outcome indicators*								
Consistent condom use with occasional clients								
Collective efficacy and corresponding mediators self-efficacy for condom use with commercial partners								
Low	Ref	Ref			Ref	Ref		
High	0.9 (0.7–1.4)	6.3 (4.5–8.9)	6.8 (4.3–11.0)	0.000	3.1 (2.3–4.2)	1.9 (1.4–2.6)	0.7 (0.4–1.0)	0.061
Collective agency and corresponding mediators self-efficacy for condom use with commercial partners								
Low	Ref	Ref			Ref	Ref		
High	0.5 (0.4–0.7)	8.0 (4.7–13.6)	14.9 (8.2–27.0)	0.000	3.3 (2.4–4.4)	1.2 (0.9–1.7)	0.4 (0.3–0.6)	0.000
Collective action and corresponding mediators self-efficacy for condom use with commercial partners								
Low	Ref	Ref			Ref	Ref		
High	1.2 (0.8–1.9)	1.6 (1.1–2.4)	1.4 (0.8–2.4)	0.437	3.1 (2.3–4.2)	2.0 (1.5–2.6)	0.7 (0.5–1.1)	0.069
Consistent condom use with regular clients								
Collective efficacy and corresponding mediators self-efficacy for condom use with commercial partners								
Low	Ref	Ref			Ref	Ref		
High	1.2 (0.9–1.6)	3.5 (2.6–4.8)	3.1 (2.0–4.9)	0.001	3.1 (2.3–4.1)	2.0 (1.5–2.6)	0.7 (0.5–1.0)	0.073
Collective agency and corresponding mediators self-efficacy for condom use with commercial partners								
Low	Ref	Ref			Ref	Ref		
High	0.5 (0.4–0.7)	10.2 (6.9–15.2)	20.1 (12.6–32.3)	0.000	3.2 (2.4–4.3)	1.2 (0.9–1.7)	0.4 (0.3–0.6)	0.000
Collective action and corresponding mediators self-efficacy for condom use with commercial partners								
Low	Ref	Ref			Ref	Ref		
High	1.1 (0.7–1.6)	2.0 (1.5–2.7)	1.9 (1.2–3.1)	0.001	3.0 (2.3–4.0)	2.0 (1.6–2.7)	0.7 (0.5–1.1)	0.583

*AOR* adjusted odds ratios, *Ref* reference variable, *CI* confidence intervals

^a^ Odds ratios were adjusted for the corresponding mediators

^b^ Odds ratios were adjusted for the corresponding collectivization indicators with the socio-demographic characteristics: age of FSW; formal schooling (yes, no); marital status (currently married, not currently married); source of income other than sex work (yes, no); place of solicitation for sex work (home, public places, brothel/lodges); visited any place for sex work in past 2 years (yes, no); duration of sex work in years (entered as continuous variable)

## Discussion

The findings of this study indicate that the majority (more than four-fifths) of FSWs in Andhra Pradesh report a high degree of collective efficacy, reflecting the confidence that the community mobilization program has built within sex workers over time. The increase noted in collective action from 2010 to 2012 suggests that FSWs started to participate in activities that concern all or some FSWs. These findings on levels of community mobilization are similar to those observed in other studies of community mobilization interventions in India [5, 11, 16, 20, 27]. The study further adds that the marginal decline in collective efficacy and collective agency among FSWs in the inter-survey period may be due to the change in the sex work environment in the state of Andhra Pradesh. Previously published research in the same geographies suggest that there is fluidity in the ways clients are solicited by sex workers [38, 39], which could potentially change the dynamics within community mobilization programs as many sex workers become independent (self-sufficient) due to the nature of their sex work practice. This is evidenced in the current study, which shows an 11 percentage point increase during the inter-survey period in FSWs who use cell phones for solicitation of clients. With the increased use of cell phones and operating independently due to increased confidence, FSWs’ belief about depending on other sex workers (which is synonymous for collective efficacy) is likely to change and the same has been noted in the current study. Whereas, collective action is the last stage of the community mobilization program wherein empowered sex workers likely to participate in activities together with other members of the group. We note that these changes as with increased collectivization, there are considerable proportions of sex workers likely to participate in different activities includes group of FSWs coming together to demand or help other community members to access one or more of the seven entitlements (e.g. ration card, voter card, bank account, free education for children, health insurance, representation in government forums and better health services from government). Whereas, a substantial proportion of sex workers as they become more knowledgeable (as a result of program) about the processes and dealing with administration, their reliance on other sex workers might go down. The current study results reflect this transition in community mobilization of sex workers over time.

In this study, CCU with different clients/partners and self-efficacy for condom use with different clients or partners among FSWs have significantly increased over the survey period. However, these results become more relevant when they are analyzed and presented through the lens of community collectivization measures. FSWs with a high degree of collective efficacy and collective agency have shown a significant improvement in CCU with both occasional and regular clients at both the survey periods. The time and collectivization (collective efficacy and collective agency) interaction effect is significant in the study, indicating a sharp increase in CCU over time for each of the collectivization measures. In other words, the likelihood of reporting CCU with occasional and regular clients has increased respectively by six and threefold among FSWs with a high degree of collective efficacy in the inter-survey period as compared to those with a low degree of collective efficacy. Similarly, the odds of reporting CCU with occasional and regular clients have increased respectively by 14 and 19 times among FSWs with a high degree of collective agency in the inter-survey period as compared to those with a low degree of collective agency. A high degree of collective action is significantly associated with CCU with both occasional and regular clients in 2012; however, the interaction effect between time and collective action is not significant. The findings of this study further describe the role of the potential mediating factor, self-efficacy for condom use, which determines the overall effect of collectivization on the study outcome. The time and collective agency interaction effect reveals a significant improvement in self-efficacy for condom use with clients. It illustrates that the likelihood of reporting high degree of self-efficacy for condom use with clients has increased threefold among FSWs with a high degree of collective agency in the inter-survey period as compared to their counterparts. The mediating effect of FSWs’ high degree of collectivization and corresponding mediator (self-efficacy for condom use) shows a significant impact on the outcome indicators in most instances. The mediating effect of collective efficacy and collective agency on FSWs’ CCU with commercial clients increased from 2010 to 2012, even after adjusting for socio-demographic characteristics and self-efficacy for condom use; whereas the mediating effect of FSWs’ self-efficacy for condom use declined in the inter-survey period, after adjusting for socio-demographic characteristics and community mobilization measures. These findings suggest that positive behavior change is linked to strong community mobilization among FSWs in India, a phenomenon noted in previously published studies [11, 16, 20, 30].

This study extends knowledge from a previously published research study by the authors [20], by presenting the change in collectivization and condom use indicators and their association over time. The analyses may appear similar, because of the basic theory of change of the Avahan program on the relationships between community mobilization and condom use outcomes. Results in our earlier paper indicate that collectivization improves self-efficacy and self-confidence which in turn affect CCU; however, it was noted that collective action was low. Results in this paper further suggest that the relationship between collectivization and CCU not only remains stronger, but over time, with the increase in collectivization measures, self-efficacy and CCU also increase. Collective action has also shown a significant increase from the previous round. This finding has implications in terms of the continued role that collectivization has in increasing CCU behavior over time; and the role of improved collectivization in sustaining these behaviors. This is a unique contribution, and the presentation of results from the two rounds of data makes this study distinct from the previously published article. In addition, knowledge of community mobilization and other indicators at two points of time have more robust policy implications than at a single point of time.

Results of this study show that structural interventions (such as community collectivization) for HIV prevention can have both a positive and sustained impact on behavior change among FSWs. In other words, community collectivization not only enhances FSWs’ self-efficacy and self-confidence, it also ensures the continued practice of safe sex behaviors, a result that is noted from both the rounds of the survey. The challenge, however, going forward for the interventions is to continue FSWs’ collectivization in order to sustain safe sex behaviors. Results also indicate the need for stronger program efforts within Avahan, so that collective agency and collective action improve in those geographies where it is low. These results have implications for programs across the world, which implement structural interventions for HIV prevention within concentrated epidemic settings. Structural interventions being planned or being currently implemented with an emphasis on community mobilization globally [4, 20, 23, 27–29] must recognize the process of change in collectivization and its outcomes. Analyses of the two rounds of BTS presented in this paper also offer a theory of change framework for program planners to the extent which prevention programs include the community mobilization initiatives and the transition over time. Although, the study offers important implications, the findings may be interpreted in the light of certain limitations. First, all the independent, mediating and outcome indicators were based on self-reports, which are vulnerable to recall and social desirability biases. Second, the outcome indicator was based on only one item, which may have validity issues. Third, the analyses are cross-sectional and causality cannot be assumed as in the case of prospective research studies. Fourth, the study can be generalized only to those areas and key populations where the Avahan program or similar interventions have been implemented.

In summary, lessons learnt from this study and previously published literature [4, 5, 10, 11, 16, 20, 24, 29–31] suggest that community mobilization among sex workers works as a mechanism to popularize and enhance safe sex practices, and build self-efficacy to demand basic rights and quality services at the ground level; however, the transition in the way sex workers perceive the benefits from other members of the sex workers group are likely to change over time. The study findings further suggest the need for community mobilization programs to recognize this transition, and make necessary adjustments to sustain the confidence among sex workers groups to help each other in case of crisis. As most HIV prevention programs in India, including Avahan, are in the transition phase to the government’s National AIDS Control Program, more multilevel operational approaches are required to stabilize collectivization measures to sustain HIV reduction in the country.
